# Developing sustainable career paths as teacher-researchers in a changing academic landscape: A tale of two EFL teachers in China

**DOI:** 10.1371/journal.pone.0285363

**Published:** 2023-05-04

**Authors:** Hua Lu, Xiaorong Zhang

**Affiliations:** 1 School of Foreign Studies, Anhui Polytechnic University, Wuhu, China; 2 School of Foreign Studies, Anhui Normal University, Wuhu, China; Tallinn University: Tallinna Ulikool, ESTONIA

## Abstract

**Purpose:**

The study aimed to investigate how two English as a Foreign Language (EFL) teachers integrated their teacher and researcher identities to achieve sustainable professional growth in the context of a changing academic landscape.

**Methods:**

Using purposive sampling, two EFL teachers from a non-elite public university in China were selected as participants for this qualitative study. Data were collected and triangulated from multiple sources, including semi-structured interviews, narrative frames, document analysis, and the academic profiles of the participants. A qualitative, inductive thematic approach was employed in the data analysis. Adopting “identity” as an analytical lens, this study examined how the two participants underwent different identity trajectories to develop into teacher-researchers under the influence of their personal values and beliefs, as well as contextual factors such as institutional research policy.

**Findings:**

Throughout their identity construction process, the two participants encountered identity deficits and tensions between multiple professional roles, leading to their struggles with identity and complex identity (re)construction. While the participants underwent interactions between various forms of identity throughout their careers, they enacted agency to take actions and draw on available resources to address their identity deficits and conflicts, ultimately resolving them by pursuing a sustainable career path as teacher-researchers in their situated socio-institutional environment.

**Conclusion:**

Despite their different identity trajectories, the construction of the participants’ teacher-researcher identity facilitated their continuing professional development. This study contributes to our understanding of the complexities of EFL teachers’ identity (re)construction when seeking to establish sustainable career paths in a changing academic context. This study also has implications for both EFL academics and university management on how to assist EFL teachers in integrating teacher and researcher identities to achieve sustainable professional development in higher education.

## Introduction

There has been a surge of research on EFL teachers’ professional identities in higher education over the last two decades [1–4]. Scholars have investigated numerous topics, such as the formation of EFL teacher identities [1, 2], challenges in EFL researcher identity construction [5], and EFL teachers’ identity transitions in educational settings with reforms and managerial practices [6]. Previous studies have shown that exploring EFL teachers’ professional identities can lead to a better understanding of their professional practices and offer implications for facilitating their career advancement and personal and professional well-being [3, 6, 7].

In recent years, given the current wave of educational reforms with an emphasis on research excellence [8, 9], increasing attention has been paid to EFL teachers’ researcher identity as an integral part of their professional identities [10–13]. In many educational contexts involving a large number of EFL learners, EFL teachers have been primarily recruited based on their language proficiency with the role of English language instructors or language service providers to other disciplines [14], with their primary task being to teach students’ English language proficiency while dealing with heavy teaching workloads [13]. EFL teachers’ primary professional role is as a teacher, and their role as researchers is undervalued in their recruitment [15]. However, with the prevalence of the “publish-or-perish” culture [16], managerialism [17], and performativity system [18] in today’s higher education, university academics are all under mounting pressure to meet socio-institutional demands regarding their research output in quantifiable indicators [8]. EFL teachers are no exception. Even though EFL teachers are severely disadvantaged in conducting research [19] due to their marginalized academic status with a weak research tradition [15], they are now socio-institutionally compelled to take on the role of a researcher [19] if they are to survive and thrive in the regime of managerialism in higher education.

Therefore, while EFL teachers continue to serve as English language teachers with pedagogical practices [14], they also need to develop a researcher identity since their promotion, contrast renewal, and career advancement are all heavily reliant on their ability to meet research demands in the current higher education context [7, 11]. However, such a research-oriented reconstruction of professional identity may cause identity disorientation and tensions in traditionally teaching-focused EFL teachers [15]. Previous research has found that while some EFL teachers may lack strong intrinsic motivation [20] to transition from teacher to researcher, others may pursue research excellence at the expense of teaching quality [21]. Moreover, university teachers’ identity tensions and conflicts may be exacerbated by the intensified “publish-or-perish” syndrome in higher education [22]. In Tran, Burns, and Ollerhead’s [12] study, English language teachers responded differently to a new institutional research policy: only a few showed enthusiasm for the changing researcher role, while the majority expressed pressure and discontent in the development of their academic identity. In a similar vein, a group of university EFL teachers went through a dynamic process of identity negotiation with a research dimension to reconcile new expectations and competing demands in the managerial context of higher education [19]. According to Yang, Shu, and Yin [23], the difficulty in integrating teacher and researcher identities in the competitive higher education environment led to identity tensions among EFL teachers accompanied by negative emotions such as powerlessness and anxiety.

Prior research has also emphasized the importance of agency in assisting EFL teachers in reconstructing their professional identities in changing higher education contexts. For example, in Yuan’s [24] study on the research journey of an EFL teacher, the participant constantly exerted agency to negotiate his professional identities with various contextual factors, such as institutional research policy, eventually becoming a novice researcher in the “publish-or-perish” academic culture. Through a longitudinal case study of an English language teacher in Hong Kong, Huang [25] discovered that while personal and social-institutional factors all exerted influence on the participant’s becoming and being an English teacher, teacher agency played a critical role in constructing her professional identity and facilitating her professional development. Similarly, Nakata, Tokuyama, and Gao [26] found that an English language teacher in Japan developed her agency while striving to integrate motivation strategy research into teaching practice, and became an agent of motivational strategies. As Teng [27] noted, a sense of agency is necessary for EFL academics to cross the boundaries of different academic communities and assist them in sustainable identity development in situated settings.

Despite a growing body of studies on the professional identities of EFL teachers, limited attention has been paid to their sustainable professional development through the integration of teacher and researcher identities in the changing higher education context. As research has become a central dimension of EFL teachers’ professional work [7], it is meaningful to explore how EFL teachers integrate researcher identity with their teacher identity to facilitate their sustainable development in socio-institutional settings. Against such a backdrop, the present study, adopting “identity” as an analytical lens [28], examines how two EFL teachers developed their sustainable career paths as teacher-researchers in light of their storied experiences in the higher education context in China. “Identity” refers to “the way we make sense of ourselves and the image of ourselves that we present to others” [29] (p. 48). Derived from the dynamic interactions between individuals and their sociocultural contexts [30], identity can be used as a theoretical framework to help us better understand the psychological state of teachers by considering how they perceive and present themselves and how their perceptions influence their professional development in specific settings.

Following the definition of identity, teacher identity can be explained as teachers’ understanding of “how to be” and “how to act” with professional commitment [31] (p. 15). Identity holds a central position in teachers’ professional development [32], given that it assists teachers in positioning themselves, setting personal and professional goals, and determining the direction of efforts in terms of professional growth [33]. As a complex, dynamic, and multifaceted construct [34], teacher identity is influenced by various sociocultural, institutional, and personal factors [35], such as institutional policy and resources [12], individuals’ personal experiences, motivations, and engagement [36], and their interactions with colleagues, students, and the wider academic community [10]. Identity development is continually evolving and ongoing as teachers reflect on and adapt their practices in their specific socio-institutional context.

Given the dynamic and fluid nature of teacher identity [34] and the changing higher education contexts in which they are embedded, university teachers may adopt various identities in their professional work, which may not always be in alignment with each other [36]. Through teachers’ daily practices and social interactions, university teachers’ professional identities can be demonstrated in a combination of actual, ideal, and ought identities [37]. Actual identities are who university teachers are at the moment; ideal identities are who they aspire to become; and ought identities are the externally required roles that university teachers have to fulfill [37]. Among these, teachers’ ideal identities represent their personal orientations regarding values and goals, which influence their professional practice and ongoing development [36]. However, due to various influencing factors, there may be a misalignment between teachers’ ideal identities and their ought identities. As a result, teachers may need to agentively and actively navigate contextual demands and their ideal identities and bridge the gap between their actual and ideal identities.

In the dynamic academic landscape of higher education, integrating teacher and researcher identities has become necessary for EFL academics’ long-term sustainable professional growth. In such a context, this study aimed to explore how EFL teachers develop into teacher-researchers capable of transcending the traditional role of language instructors and identifying with the empowered role of researchers to facilitate their sustainable professional development. The findings of this study can not only assist English language teachers in similar EFL contexts in reflecting on their experiences of career sustainability and professional well-being and finding useful ways to integrate their teacher and researcher identities, but also provide practical implications for higher education administrators and leaders on how to create a supportive institutional culture to promote EFL teachers’ continuing professional growth. One central question guides the present study: *How do EFL teachers develop their sustainable teacher-researcher identities in a changing higher education context?*

## Methodology

### Research context and participants

The study was conducted at a public, non-elite university with a middle national ranking in the central part of China. Non-elite universities such as this one, which does not belong to either Project 985 or Project 211 (two projects launched by the Chinese government aiming to boost the research output of elite universities), constitute the majority of higher education institutions in China [38]. The university was chosen as the research site for its representativeness of non-elite universities in China and its accessibility, feasibility, and familiarity [39]. Despite the fact that it is not a research-intensive university with an elite reputation, the university has adopted a research-oriented institutional culture, including stringent research requirements in faculty promotion, annual faculty assessment, and three-year key performance appraisal, with the goal of improving its national ranking and advancing its development. In addition, with the release of China’s new national research policy of breaking the “Five-Only”, which aimed to deemphasize the priority set on research in higher education [40], the university began to include demands on teaching performance and educational research grants and publications in its evaluation system to promote both teaching and research.

Using purposive sampling [41], two EFL teachers at the research site were selected as participants for three reasons. They were experienced EFL teachers with over ten years of teaching experience, which may reflect their expertise in long-term sustainable professional development, as expertise is frequently associated with years of experience and practice [42]. Additionally, both participants were actively engaged in teaching and research practices that resulted in accomplishments. For example, they both had successfully applied for provincial educational research grants, won prizes in provincial or national teaching contests, and tutored students to win prizes in various English language proficiency competitions. Finally, because of their friendly relationships with the authors, both were willing to provide rich information on their teacher-researcher identity construction, which helped ensure the completion of data collection [43]. The pseudonyms Kevin and Susan, were used to protect the participants’ privacy. The participants’ biographical information is presented in Table 1. Before the study began, research ethics approval was sought from the School of Foreign Studies at the research site, and written informed consent was obtained from the two participants in August 2021.

**Table 1:** Biographical information of the participants.

Participants	Gender	Age	Degree	Professional title	Research area	Years of work
Kevin	Male	Mid-40s	Master’s degree	Associate professor	Digital technology in education	22
Susan	Female	Late-40s	PhD	Associate professor	Cultural translation	14

### Data collection

Data were collected and triangulated from a variety of sources, including narrative frames, semi-structured interviews, institutional policy documents, and the participants’ academic profiles. The duration of data collection spanned an entire academic semester, commencing in September 2021 and concluding in December 2021. The participants were given a narrative frame at the start of the study (September 2021) to elicit general information about their backgrounds and experiences as university EFL teachers [44]. The narrative frame was adapted from Teng’s [45] with several sections, including the participants’ general background, experiences of becoming a university EFL teacher, roles related to their professional identity, and comments on their professional identity.

After eliciting general information from the narrative frames, the first author conducted one-on-one semi-structured interviews with the participants face-to-face at their convenience. The interview with Kevin was scheduled in October 2021, whereas the interview with Susan was conducted in December 2021. At the beginning of the interviews, the first author asked some individually tailored questions based on the participants’ responses in the narrative frame to gain a more thorough understanding of their experiences and perspectives. Then, the interview moved on to the participants’ work experience as university EFL teachers and their ongoing professional engagement. The participants were asked to share their beliefs about English language teaching and research and how they practiced in accordance with those beliefs. They were also invited to reflect on the possible challenges and conflicts in their professional work, their responses to such challenges, and the critical incidents in their professional lives. In the interviews, the first author paid particular attention to the critical incidents in the participants’ lived experiences, probing further questions about the impact of such incidents on their professional identities and continuing professional development. Participants were also invited to share their thoughts on the socio-institutional demands on academics’ work performance and the impact of such policies on their professional practices and future plans. Each interview was conducted in Chinese (the interviewer’s and interviewees’ mother tongue), lasted approximately one hour, was audio-recorded with the participants’ permission and was transcribed verbatim for the data analysis. After the interviews, the transcripts were returned to the participants for accuracy checking.

In addition, institutional policy documents and academic profiles of participants were collected to provide additional information and triangulate the data. With the permission of the dean of the School of Foreign Studies, institutional documents on promotion, annual assessments, and three-year key performance appraisals were collected from the research site’s website. The academic profiles of the participants were gathered from the website’s faculty introduction with the participants’ permission.

### Data analysis

A qualitative, inductive thematic approach [46] was adopted for the data analysis since we continuously moved among the data, the research question, and the analytical lens of identity. First, we repeatedly read the participants’ narrative frames and interview transcripts to familiarize ourselves with the data. Second, when we reviewed their narrative frames and interview transcripts, we paid particular attention to the words and phrases that the participants used to describe themselves in terms of professional identity and how these identities interacted with one another in their professional work. The analysis resulted in a range of themes regarding the participants’ identity construction, such as “an English language proficiency teacher”, “a competent university EFL teacher”, “a novice EFL teacher with limited knowledge of language teaching theories”, “a teaching-focused EFL teacher with limited research capacity”, “an EFL teacher with no research capacity and limited teaching experience”, “an EFL teacher with research capacity”, “a competent department administrator, EFL teacher, and researcher”, “a caring EFL teacher with research expertise”, and “a conscientious EFL teacher-researcher”. Then, these identified identity themes were further compared and integrated by referring to the theoretical lens of identity to reveal the actual, ought, and ideal identities of the participants in their professional practice. For example, after realizing his lack of research capacity as a teaching-focused EFL teacher (his actual identity), Kevin tried to include the dimension of research in his professional work and become a competent EFL teacher with research capacity (his ideal identity). For Susan, she was appointed Director of the Master of Translation and Interpreting (MTI) Center at her department, which brought her an external identity as an administrator (her ought identity). Next, the identified themes of identity were further examined to analyze the possible interactions, particularly regarding identity deficits and conflicts, between one another in the participants’ work context. For instance, Kevin experienced identity tensions between his ideal identity (a competent EFL teacher with research capacity) and his actual identity (a teaching-focused EFL teacher with no research capacity), which prompted him to pursue his master’s degree at an elite university.

Furthermore, data from the institutional policy documents and the participants’ academic profiles were reviewed to triangulate with the narrative frame and interview data and to enrich our understanding of the participants’ professional identities and interactions in identity construction. For example, additional information about the stringent research requirements imposed on participants by their institution was elicited through a review of institutional policy documents. Important events listed on the participants’ academic profiles provided data that further evidenced their efforts to change their ideal identity (competent EFL teacher-researcher) into their actual identity. To enhance the trustworthiness and validity of the study, we used both data and researcher triangulation. The first author and the second author independently analyzed the data, followed by several rounds of discussion on the disagreements on the coding results, and eventually reached a consensus on the data analysis results.

## Findings

This section presents chronological accounts of the two participants’ teacher-researcher identity (re)construction experiences case by case to shed light on the complex and ongoing development of their sustainable career paths in situated contexts.

### Kevin’s story

Kevin was a veteran EFL teacher with twenty-two years of experience. After graduating from a prestigious normal university in central China in 1999, he was drawn to “the simple life on campus with a focus on teaching” (narrative frame) and thus became a university EFL teacher (academic profile).

Kevin was assigned several English courses after joining his work institution, ranging from oral English to academic English writing. Such responsibilities demonstrated his ought identity as a university EFL teacher with primary responsibility for teaching students’ English language proficiency imposed by socio-institutional expectations. Although Kevin was a novice English teacher at the time, he believed he could develop his identity as “a competent university EFL teacher” (his ideal identity) (interview). First, he was eager to learn different teaching methods and to get to know his students well (narrative frame); second, he spent a significant amount of time preparing teaching courses and teaching several courses intensively for several years, which helped him accumulate rich teaching experience; third, he actively tutored students to participate in various English language proficiency competitions and had fruitful results. The combination of these not only gave him a sense of accomplishment and pride but also aided in the development and nourishment of his budding identity as a conscientious and committed university EFL teacher.

*As a university EFL teacher*, *I believe that the accomplishments of my students are my accomplishments*. *What I value most as a teacher is not the students’ academic achievements; rather*, *I value cultivating students to be useful talents to society*. *Then I believe that such an accomplishment is also a kind of gain for the teacher*. (Kevin, interview)

Practicing such a teaching belief, Kevin felt that he needed to be “a teacher that can accompany students throughout the entire process of education, growth, and development” (his ideal identity). However, as a novice EFL teacher with limited knowledge of language teaching theories, Kevin encountered some challenges in living up to his ideal identity as a competent university EFL teacher. He found that his few years of teaching experience were insufficient to support his continuing professional development. After five years of repetitive and monotonous English language proficiency teaching, he had reached “a bottleneck” in his teaching without the theoretical guidance provided by research.

*Back then*, *I was simply teaching without conducting research*, *and I reached a point where I felt there was a bottleneck*, *that is*, *there was not much to teach students*. *Yes*, *we teach English language skills*, *but we may also need to study the essence of language*, *some thinking behind the language*, *or some problems in this area*. (Kevin, interview)

Therefore, although Kevin could “prepare courses and teach students English language skills and usages” (interview), he fell short of generating courses involving an in-depth analysis of the English language. Furthermore, the implementation of a research-oriented policy at his work institution exacerbated the tension between his actual identity as “an English language proficiency teacher” and his ought identity as “a competent university EFL teacher with research capacity”. A review of institutional policy documents confirms that the university imposed stringent requirements for research outputs in promotion and demonstrated a preference for awarding research-productive faculty in annual evaluations.

As a result, Kevin, a university EFL teacher with no research capacity and limited teaching experience (his actual identity), exerted agency to develop his research capacity to escape his predicament and advance his career. He pursued a master’s degree at a prestigious university belonging to Project 211 in a nearby province in 2005 (academic profile). During his postgraduate studies, Kevin quickly identified his research area, digital technology in education, and by following in his supervisor’s footsteps, learned research methodology in applied linguistics. He developed collaborative research experience in paper publications with his supervisor and peers. All of these gains gave Kevin strong confidence as a researcher in applied linguistics (his ought identity) and eventually changed his mind about research.

*Prior to conducting research*, *I felt it was quite far away*. *It was all very strange to me*. *Other people’s papers were difficult to understand*, *and I thought research was sacred*, *a high-level thing at the time*. *Now*, *I have a better understanding*. *Research is no longer as far away as it once was*. *It is something that is around me*, *similar to my teaching*. (Kevin, interview)

By exercising agency to address the problem of lack of research capacity, Kevin managed to alleviate the tension between his actual identity (a teaching-focused university EFL teacher with limited research capacity) and his ideal identity (a competent university EFL teacher in both teaching and research). In addition, to produce sufficient research outputs, Kevin exerted agency to actively apply for research grants and write manuscripts. The institutional policy demonstrated that these two requirements were evident in every research document. Given Kevin’s view of promotion as “a baton”, his efforts were aimed in that direction.

*The promotion system is a baton for all teachers*. *One is that it pushes teachers to conduct research*, *which is something we must do*. *Second*, *it requires that our research outputs not deviate from our professional field*. *So*, *it may impose some constraints on us*. (Kevin, interview)

Kevin’s consistent effort in research practice resulted in several provincial and institutional research grants and over ten paper publications. He was promoted to associate professor in 2014 (academic profile). Since, he has held several administrative positions, including Director of the English Department and Secretary of Research at the School of Foreign Studies. However, his institutional expectation as a competent department administrator, EFL teacher, and researcher involved multiple responsibilities, which led to Kevin’s identity struggles.

*The administration consumes a lot of energy*, *which diverts my attention away from research or teaching*. *For example*, *we must submit a large amount of paper work at the start of the term*, *followed by a mid-term inspection form*, *and finally a completion form*. *We have to submit a lot of materials repeatedly*, *which is very troublesome*. (Kevin, interview)

Kevin had to put his researcher identity on hold for several years due to time and energy constraints and “slackness after promotion” (interview). Then, in around 2020, as his institution began to implement China’s new research policy breaking the “Five-Only”, both teaching and research were given stringent requirements in the key-performance appraisal system. Therefore, Kevin had to reclaim his identity as an active researcher despite his struggles and frustrations.

*I haven’t done much reading in the last few years*. *So*, *when I started to do research again*, *there were a lot of things I needed to catch up on*. *As with applying for research grants*, *my lack of preparation may result in poor results*. (Kevin, interview)

Unsurprisingly, when Kevin rushed to apply for a provincial research grant without doing much reading or preparation, he failed. After the initial painful feelings of picking up research activities, Kevin realized that he could integrate teaching and research. “Teaching can provide inspiration for research, and research can promote teaching effects” (interview). Kevin shared his memorable experience of conducting empirical studies in class with us.

*It took me a significant amount of time to design research*, *enter data into a computer*, *and compare the results of several classes*. *I spent a lot of time in front of the computer every day*, *especially when it came to data processing*. *It took a lot of time and effort on my part*. (Kevin, interview)

As a result, while Kevin was under mounting pressure to address identity tensions and institutional demands on his work duties, his agency in integrating teaching and research has assisted him in finding a practical and effective solution to contextual challenges. In the last three years, he has not only had four papers on educational research published in international peer-reviewed journals, but he has also been named “excellent teacher” at his institution twice because of his high rankings in students’ evaluations of teachers’ teaching performances (academic profile). Such professional gains demonstrate Kevin’s achievements in both teaching and research, as well as his sustainable professional development, given that his integration of teacher and researcher identities has met both his personal needs and institutional expectations. Being a university EFL teacher and a researcher are two crucial roles for Kevin (narrative frame). As he shared,

*Teaching and research*, *in my opinion*, *are like stairs*. *When we teach*, *we gradually improve our teaching level*, *and once that level has improved*, *we will have a research platform*. *Then*, *as our teaching level improves*, *so will our research platform*. *As a result*, *teaching and research progress together*. (Kevin, interview)

Overall, Kevin joined his university with an actual identity as an English language proficiency teacher, and he made agentive efforts to overcome identity tensions (e.g., “a teaching-focused university EFL teacher” versus “a university teacher with research capacity”) and live up to his ideal identity as a competent university EFL teacher in both teaching and research. Over time, his agency-driven actions enabled him to integrate teaching and research, ensuring sustainable professional development in his situated work context. **Susan’s story**

Unlike Kevin, who joined the university with a bachelor’s degree, Susan joined the university after completing her master’s degree in 2008. Susan quickly discovered that the university had adopted a research-oriented institutional culture and placed a high value on faculty research output, which provided her with an external identity as a university EFL teacher with a research dimension (ought identity), as reflected in the university’s research requirements for newly recruited faculty such as Susan.

*The requirements of my university for my professional practice are both teaching and research… In research*, *two high-level papers in three years are required*. *I will be given certain awards if I meet these requirements*, *like 2*,*000 RMB for each paper*, *or I will suffer a financial loss if I fail*. (Susan, narrative frame)

Under such influence, Susan made conscientious efforts in research practice, focusing on developing her researcher identity. In her spare time, she “frequently read academic literature” and “frequently conduct(ed) research” (narrative frame). Susan prioritized her researcher identity over her teacher identity during the first few years of her career, as evidenced by her comments.

*When we call a person a teacher*, *it’s only limited to teaching*. *But if we want to be researchers*, *we must improve ourselves and then keep moving forward so that we can become researchers*. *I think the researcher is better than the teacher*, *and the level is higher*. (Susan, interview)

Despite her dedication to research, Susan discovered that her limited training during her master’s degree did not provide her with the necessary skills to progress in research. There was a disconnect between her ought identity (an EFL teacher with research capacity) and actual identity (an EFL teacher with limited research knowledge and skills). Therefore, she made a decisive move in 2012, enrolling in a doctoral program at a prestigious university in Shanghai (academic profile), which proved to be a critical event in her research career.

*These three years have been beneficial to me*. *I was at a university in Shanghai at the time*, *and there was a strong research atmosphere there*. *The scholars and their profound ideas there were very inspiring to me*. *Also*, *my supervisor had led me very well; under his guidance*, *I had identified my research area*, *American religion and culture*, *and I had collaborative work with my supervisor as well*, *so I believe that was the start of my research*. (Susan, interview)

As a result of her consistent research efforts and acquired research knowledge and skills during her PhD studies, Susan was awarded a research project funded by China’s National Social Sciences Foundation in 2016 and quickly promoted to associate professor (academic profile). Despite her research accomplishments, Susan considered herself a “beginner” in her field (actual identity). She looked up to some experts in her field and aspired to be an established researcher like them (ideal identity). Therefore, when she returned from Shanghai, she concentrated on furthering her research in her area, American religion and culture, while continuing to teach college English routinely. Her primary focus on research and relative ignorance of teaching revealed an identity deficit in her professional work. She was only attempting to fulfil a prescribed duty (research engagement) associated with her ought identity as a university teacher with research capacity (externally advocated by the university), with comparatively few efforts to utilize expertise associated with such an identity in her teaching practice.

Things changed when Susan was appointed Director of the Master of Translation and Interpreting (MTI) Center at her department in 2018 (academic profile). Susan’s department imposed a new external identity as an administrator (ought identity) on her. Surprisingly, Susan held a positive attitude toward her newly imposed identity.

*Although being Director of the MTI Center makes my daily work busier*, *I think it is a better platform*. *It provides me with more opportunities to meet experts in the field and see a wider world*. (Susan, interview)

Susan’s professional engagement with the MTI Center became an important turning point in her identity transformation. First, Susan’s attitude toward teaching has shifted due to her frequent communication with postgraduate students. Through the ongoing process of teaching translation courses to postgraduate students, Susan found that she enjoyed being a teacher.

*I want to provide these postgraduate students with high-end content*, *so I sometimes share my research with them in class*, *and when I share*, *it gives me a kind of happiness*. *Furthermore*, *when I am with these young students*, *I feel very energetic and alive*. *This simple and routine life suits me*. (Susan, interview)

Second, Susan’s recent years of postgraduate teaching experience in translation and culture gave rise to her identity as an “insider” with knowledge about students’ needs, which further assisted her in reducing her identity deficit and contributing to the internalization of her ought identity as a teacher-researcher. Susan felt the need to become a teacher-researcher (ought identity) after several years of teaching postgraduate courses, as well as her institution’s implementation of breaking the “Five-Only” policy with an emphasis on both teaching and research. Therefore, Susan attempted to shift her research focus from American religion and culture to cultural translation to better connect her research expertise to the needs of her students. Susan’s transition was a success in both her research and teaching. She published one translation book last year and received two institutional educational research grants based on her teaching experience in cultural translation (academic profile), which helped her realize her identity as a teacher-researcher.

*I had not anticipated receiving both of these educational research grants*. *I got them*, *much to my delight*. *Perhaps because I have combined what I have learned over the years with teaching experience*, *and my ideas are appropriate for this type of educational research*. *Anyway*, *it’s a good motivation for me to incorporate teaching reflection into my research practice*. (Susan, interview)

In teaching, Susan tried to actualize her research experience and expertise in cultural translation into teaching practice. As Director of the MTI Center, Susan felt responsible for “promoting students’ translation skills” (interview); she invested great time and energy in her coursework and various kinds of translation practice practicums to fill her identity deficit and get close to her ideal identity (a competent and caring EFL teacher with research expertise). Based on her experience translating a book, Susan taught students how to “understand the overall framework of an article” (interview) before translating it in detail. She has also actively led students to participate in translation workshops and competitions and created a public website to promote their translation work.

As shared by Susan, her students responded positively to this theory plus practice teaching approach, demonstrating that they “acquired translation knowledge and gained translation skills” (interview) through courses and practices. Susan gained confidence in becoming a competent EFL translation teacher with a research dimension (ideal identity) as a result of the students’ positive responses. She expressed a desire to continue carrying out such a practice.

*I want my students to have knowledge of translation theory as well as practical translation skills*. *We recently translated our city’s publicity brochure*, *as requested by the government*. *All of the students who took part felt great about practicing their translation skills*. *This is excellent practice for them*. *We will do so as long as opportunities present themselves*. (Susan, interview)

Susan’s identity has shifted from a university teacher with research capacity (her ought identity) to that of a conscientious teacher-researcher (her actual identity) over the last few years as Director of MTI Center, which has facilitated her sustainable professional development in both teaching and research. Such a shift in her professional identity was evident in her drawing on research experience and expertise in cultural translation to educate her students. In other words, her researcher identity influenced her teaching practice and teacher-researcher identity development. As Susan reflected,

*Research aids teaching because it requires us to read more books and experience things firsthand*, *which gives us a broader perspective*. *In my case*, *I translated a book*, *which provided me with valuable experience to share with students*. *Teaching is a guide for students*, *and researchers can lead them to a higher level*, *so I believe research is essential for teaching*. *We should be researchers if we want to be good teachers*. (Susan, interview)

Susan began her work with a researcher identity, which evolved into an identity deficit as she prioritized research excellence over teaching responsibilities. She gradually resolved her identity deficit by exercising agency in linking research with teaching practice as she engaged in continuing professional development with new responsibilities. Her actual identity as a teacher-researcher served as a guide for her sustainable professional development in the context of higher education.

## Discussion

Using “identity” as an analytical lens, this study discovered that participants had a variety of ought, actual, and ideal identities [37] before becoming teacher-researchers for their sustainable professional development. The findings show that the professional identities of EFL academics are dynamic, complex, and multifaceted in nature [34]. The participants’ eventual teacher-researcher identities emerged from the multiple identities they had constructed in response to their personal values, beliefs, and socio-institutional contexts.

Subject to various internal and external factors [35], the participants’ multiple identities (ought, actual, and ideal) frequently align or misalign [36], sometimes resulting in identity tensions or conflicts. These identity tensions influenced academics’ understanding of their profession and caused them to constantly (re)construct their professional identities. For instance, Kevin started his career as a novice EFL teacher, and then attempted to integrate language learning theories with teaching practice in his classrooms through research excellence pursuit, suggesting a sense of alignment between his actual identity (an EFL teacher with limited research knowledge) and ideal identity (an EFL teacher with research capacity). However, such identity alignment was later disrupted due to his slackness following promotion and an externally imposed administrator identity by his department, resulting in conflicts between his ought identity (a competent department administrator and EFL teacher) and ideal identity (a committed EFL teacher-researcher). To address the conflicts between his multiple identities and sustainable career development, Kevin reclaimed his researcher identity in accordance with changes in institutional research policy.

However, this study discovered that adopting a researcher identity alone could not ensure EFL academics’ sustainable career advancement, despite the fact that becoming a researcher has been a necessity for EFL academics in the competitive higher education context [19]. As reported by previous studies [13, 20], EFL academics had a mix of intrinsic and extrinsic motivations for research engagement. In Kevin’s case, he wanted to improve the teaching effect as well as obtain promotion through research practice. Susan, on the other hand, initially prioritized researcher identity over teacher identity and later attempted to incorporate her research expertise into teaching. Susan’s early embrace of research and complete dedication to developing a researcher identity, as unusual as it was, was most likely related to the research-oriented institutional culture when she joined the university [7, 8, 11]. Academics tend to exhibit a range of emotional responses to research policies and emotion-related identities [12]. In response to the result-oriented research policy, Susan adopted a pragmatic mindset and preferred research over teaching [21]. Both participants, however, experienced an identity deficit and could not make further sustainable progress in their careers when they reached a new stage with new responsibilities (for Kevin, it was the changes in institutional policy with an emphasis on both teaching and research; for Susan, it was her new appointment as Director of the MTI Center). The identity deficit between their actual and ought identities was not solved until they integrated the teacher identity with the researcher identity [23].

It is worth noting that despite their similarity in eventual teacher-researcher identity construction, the participants displayed different identity trajectories. Kevin began his career with an identity deficit between his actual identity (a university EFL teacher) and his ought identity (a university EFL teacher with research capacity), and then progressed to teacher-researcher over time. Susan, on the other hand, began with a researcher identity, experienced an identity deficit between her actual identity (an EFL teacher with administrative duties) and her ideal identity (an EFL translation teacher with research capacity) in the mid-stage of her career, and then gradually embraced her ought identity as a teacher-researcher. Such differences can mainly be attributed to their personal values and beliefs and individual experiences in situated contexts [10, 22, 35]. Echoing the findings of previous studies [14, 15], Kevin, a teaching-focused EFL teacher, demonstrated his value of teacher identity over researcher identity. His construction of a researcher identity was partially due to his desire to guide his teaching practice with theories. After being promoted to associate professor, his external motivation vanished [20], and he sidelined his researcher identity until compelled to reclaim it by a new institutional research policy. Susan, however, was devoted to the pursuit of researcher identity, believing that research was at a higher level than teaching. Later, she was assigned new responsibilities that were directly related to the overall teaching quality of postgraduate students, giving rise to a new ideal identity (a competent translation teacher with research expertise) and requiring her to invest efforts in integrating her research expertise with teaching practice to have sustainable career development. This finding indicates that, as a result of their individual values and ongoing practice, EFL academics’ actual, ought, and ideal identities are not static and may change over time [34].

While EFL academics’ professional identity construction is permeated with numerous changes and identity conflicts, such changes and conflicts can also present opportunities for them to exercise self-agency, allowing them to reshape their professional identities and advance their sustainable career development. Consistent with the findings of previous studies [24–26], this study revealed that agency played an important role in EFL academics’ actions in bridging their identity deficit. In Kevin’s case, he experienced conflict between his ought identity (an EFL teacher with research capacity) and his actual identity (an EFL teacher with limited research knowledge and skills) in the early stage and conflict between his actual identity (an EFL teacher with administrative positions) and his ideal identity (an EFL teacher-researcher) in the mid-stage. Both conflicts resulted in an identity deficit when he wanted to advance his career. Then, by pursuing professional development through ongoing agency-driven actions such as enrolling in a master’s program, applying for research grants, writing manuscripts, and incorporating research experience into teaching, he gradually constructed his ideal identity as a teacher-researcher and filled his identity deficit. For Susan, the conflict between her ought identity (a translation teacher with research expertise) and her actual identity (an EFL teacher focused on research) made her realize that she needed to transform from her actual identity to her ought identity to solve the identity deficit. She developed new forms of professional knowledge and identity as a conscientious teacher-researcher by agentively adjusting her research area and integrating her research expertise into teaching practice, which contributed to her sustainable development in a changing institutional context. Both Kevin and Susan’s experiences testified to the importance of agency in the identity construction of EFL academics. Self-agency could help them overcome contextual constraints [27] and provide new opportunities for identity (re)construction.

## Conclusion and implications

This paper reports on two EFL teachers’ sustainable career trajectories in a changing academic environment. Given the current wave of educational reform in higher education, this study adds to our understanding of EFL teachers’ professional identity (re)construction and sustainable career development in a shifting and complex higher education context, and it may be of relevance to EFL teachers undergoing similar academic changes in other contexts worldwide. The findings shed light on the interactions between the various forms of identity within EFL teachers throughout their careers and how such interactions influence their professional development. The findings also revealed that the participants exercised their agency to take action and draw on available resources to address their identity deficits and conflicts, thereby contributing to their sustainable career paths with an enriched ideal identity as teacher-researchers.

This study has implications for both EFL teachers and university management regarding academics’ sustainable professional development in higher education. First, given the dynamic and multifaceted nature of teacher identity [34], EFL teachers need to develop the ability to reflect on and act on their identity deficits and conflicts in situated contexts. For example, when EFL teachers notice an identity deficit or conflicts between their multiple identities at work, they can reflect upon how their identities interact with one another, what caused their identity conflicts, what challenges such interactions and causes bring, and what professional identity they hope to become by addressing the challenges and conflicts. Such reflections may not only assist EFL teachers in better understanding the corresponding demands on their professional identity in a changing academic context but may also encourage them to take agentive actions to turn identity conflicts and challenges into opportunities for further professional development. As demonstrated by the cases of the participants, Kevin pursued his master’s degree to resolve his identity deficit by becoming a researcher, whereas Susan transformed her identity conflict between a research-oriented teacher and a teaching-focused translation teacher by agentively adjusting her research area to integrate teaching and research practices.

Second, the different identity trajectories of the participants suggest that university management, such as university administrations and policy-makers, should provide sufficient contextual support to EFL teachers in their identity (re)construction. Kevin taught for several years and only acquired research knowledge and skills when he attending an elite university for his master’s degree. It is therefore suggested that a research mentoring system at the departmental level could be provided to novice EFL teachers to raise their research awareness and hone their research skills from the start of their careers. Susan only became committed to her teaching responsibilities when she was appointed Director of the MTI Center after several years of work experience. Thus, university management may also consider providing teaching-practice-based programs to instill a sense of teaching commitment in beginning EFL teachers, particularly those novice teachers who began their careers influenced by the research-oriented academic culture. In both the research mentoring program and the teaching-practice program, EFL teachers can meet regularly, collectively discuss their potential identity conflicts and tensions in teaching and research practices, learn from experienced teachers and mentors, and devise coping strategies to address challenges and enhance their teacher-researcher identities.

## Limitations

This study also has some limitations. First, this study collected data for only one academic semester, which was a relatively short period. Researchers interested in the sustainable professional development of EFL teachers can conduct a longitudinal study to investigate their identity development and professional growth in a higher education context. Second, while this study used data triangulation to collect data from multiple sources, it did not collect data from other stakeholders, such as university administrators, to explore their perspectives on EFL teachers’ sustainable professional development. Future research may collect data from other important stakeholders in higher education to present a more complete picture of EFL teachers’ identity (re)construction progress.
